# The effectiveness of expressive art therapy on infertile women undergoing surgery: study protocol for a randomized controlled trial

**DOI:** 10.1186/s13063-024-08324-1

**Published:** 2024-07-12

**Authors:** Li Liu, Huiyan Yang, Anjiang Lei, Huaxuan You

**Affiliations:** 1 Department of Obstetrics and Gynecology Nursing, West China Second University Hospital, Sichuan University/West China School of Nursing, Sichuan University, Chengdu, China; 2 Key Laboratory of Birth Defects and Related Diseases of Women and Children (Sichuan University), Ministry of Education, Chengdu, Sichuan China

**Keywords:** Expressive art therapy, Infertile women, Study protocol, Randomized controlled trial

## Abstract

**Background:**

Infertility produces infertility-related stress in both members of infertile couples, especially for infertile women. Some studies verified the negative relationship between infertility-related stress and outcomes of infertility treatments. Effective mental health care during fertility treatment is urgently needed, but there has been a lack of efficient support services. To reduce the infertility-related stress of infertile women, expressive art therapeutic schemes will be organized and implemented by certified international expressive art therapists.

**Methods:**

This study is a randomized controlled trial. Participants in the intervention group will receive expressive art therapies after the baseline investigation. Expressive art therapies will be led by the certified international expressive art therapist. The interventions include progressive muscle relaxation training, music meditation and drawing therapy. Participants in the control group will receive routine care. The Hospital Anxiety and Depression Scale (HADS) and Fertility Problem Inventory (FPI) will be used to investigate the anxiety, depression, and infertility-related stress of all participants at admission and at discharge.

**Discussion:**

This study will verify the effectiveness and efficiency of expressive art therapies for infertile women. The results will provide new knowledge on mental health care strategies for infertile women.

**Trial registration:**

ChiCTR, ChiCTR2300070618. Registered 14 April 2023.

**Supplementary Information:**

The online version contains supplementary material available at 10.1186/s13063-024-08324-1.

## Background

Infertility is defined as a failure in clinical pregnancy after 12 months of regular and unprotected sexual intercourse [1]. Infertile couples with no pregnancy history are classified as having primary infertility, while infertile couples with a pregnancy history are classified as having secondary infertility [2]. Globally, the incidence of infertility among couples ranges from 3 to 25% [3–5]. In China, the incidence of infertility ranges from 15 to 25% among couples of reproductive age [2, 6, 7]. The prevalence of infertility is increasing, which might be due to the trend toward delaying childbearing [4]. As reported, infertility is a public health issue that affects the mental health, marital satisfaction, and family relationships of patients [5]. Moreover, infertility affects more women than men in terms of psychological well-being and marital relationships [8]. Traditionally, not having children is often considered a woman’s fault. This prejudice makes many infertile women suffer discrimination in their lives and bear social consequences such as violence from family, marriage breakdown, or malicious evaluation from surrounding people. Due to the traditional culture of China, infertile women may have to endure more prejudice from family and society [9].

Infertile patients experience unique psychological stress, which is known as infertility-related stress. As reported, infertile women had higher levels of infertility-related stress than men [5]. Depression and anxiety represent distress-mediated symptoms of infertility [8]. As reported, different degrees of depression and anxiety affect 50% of infertile couples, affecting their quality of life [4]. In addition, depression and anxiety were related to decreased conception rates and lower outcomes of reproductive medicine, respectively [8]. Some studies also verified the negative relationship between infertility-related stress and outcomes of infertility treatments [10]. Therefore, the mental health of infertile women requires more attention, and effective mental health care during fertility treatment is urgently needed. Previous studies reported that infertile women coping with stress might benefit by disclosing their experience of infertility [11]. To improve quality of life among infertile women, mental care was suggested to encourage the expression and sharing of emotions during treatment [12].

Art plays a significant role in health promotion, and it is a method of healing. The arts are an essential part of health and nursing care because they are beneficial in disease prevention, cure, rehabilitation and care [13]. Expressive art therapy (EAT) has been incorporated into various programs as an element of both foundational and supportive treatment. EAT combines physical and psychological nonmainstream methods, including dance/movement, drawing, painting, sculpting, music, writing, sound, and improvisation [13]. EAT provides participants of all ages with a platform to release feelings, increase self-awareness, and explore hidden feelings in a supportive setting [14]. The participants do not need any experience or skills in art making, but the expressive art therapists need them. Considering cultural differences, expressive arts therapists should be culturally competent [15]. EAT has been applied in cancer patients, psychiatric patients, neurosurgical patients, pediatric patients, and domestic violence survivors [15, 16], but very few studies have explored the effectiveness of EAT in infertile women. As reported, releasing infertility-related stress is a way to promote the mental health of infertile women, which is essential to improve the outcomes of infertility treatments. Therefore, we designed a randomized controlled trial to verify the effectiveness of EAT in infertile women. We hypothesize that EAT will decrease the levels of infertility-related stress, depression and anxiety in infertile women.

## Methods

### Study design

**Fig. 1:**
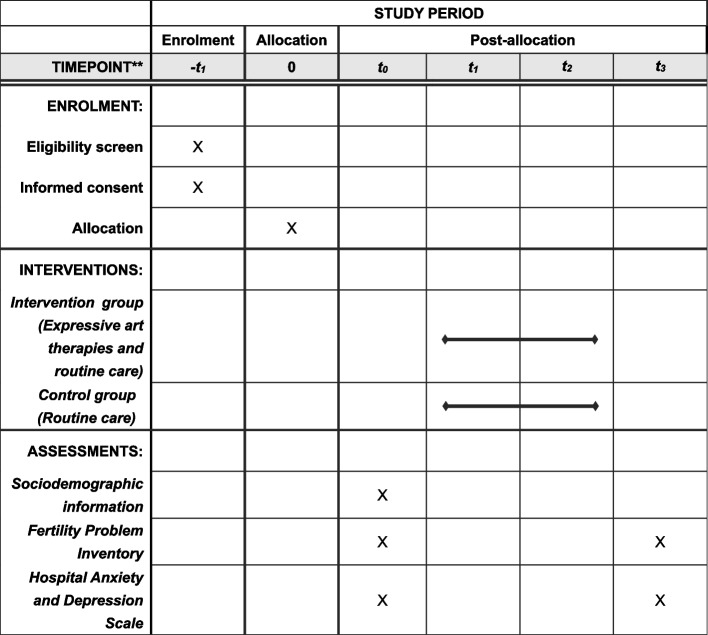
Schedule of enrolment, interventions, and assessments, according to SPIRIT 2013

This is a two-arm parallel randomized controlled trial led by an expressive art therapist. Infertile women who meet the inclusion criteria will be recruited. After providing written informed consent, participants will be assigned to either the control group or intervention group by using a random number table method. The allocation ratio is 1:1. The trial follows the Standard Protocol Items: Recommendations for Interventional Trials (SPIRIT). The schedule of enrolment, interventions, and assessments is presented in a SPIRIT figure (Fig. 1).

### Participants

Participants will be recruited from West China Second Hospital of Sichuan University, which is a women and children’s medical center in West China serving more than 5 provinces. Women are eligible if they (1) have been diagnosed with infertility, (2) will choose hysteroscopic surgery or/and laparoscopic surgery, and (3) have the ability to read and write in Chinese. Participants will be excluded if they have been diagnosed with mental illness. If participants from the intervention group and the control group are found to be sharing the same room, both will be excluded from the study to prevent any potential contamination.

### Intervention

Expressive art therapies will be led by the certified international expressive art therapist. The interventions include progressive muscle relaxation training, music meditation, and drawing therapy. The intervention time points include the first 24 h of admission (t_1_) and the first 24 h after surgery (t_2_). After baseline investigation (t_0_), participants in the intervention group will receive progressive muscle relaxation training (10 min), music meditation (10 min), and drawing therapy (40 min). The total duration of intervention is approximately 1 h. Participants in the intervention group and control group will receive general health education and routine nursing care. General health education and routine nursing care are standard practices in our hospital. By using them as the control group, we can establish a baseline for comparison, which makes it easier to evaluate the effectiveness of our intervention. Within the first 24 h after surgery, participants in the intervention group will perform progressive muscle relaxation and music meditation under guidance. At discharge (t_3_), participants in the two groups will complete the questionnaires. Participants are free to leave the study at any time for any reason without consequences. To improve adherence, all participants will be provided with guidance when they sign the informed consent form and commit to attending on the scheduled intervention time points.

### Measurement

A self-designed questionnaire will be used to collect sociodemographic information. The Hospital Anxiety and Depression Scale (HADS) is a self-reported screening questionnaire that detects mild anxiety and depression. It consists of 14 items, 7 items assessing anxiety (HADS-A), and 7 items assessing depression (HADS-D). Each item is scored on a 4-point Likert scale, ranging from 0 to 3, producing a sum score of 0 to 21 on each subscale. High scores indicate more severe symptoms [17]. The Mandarin version of the HADS was reliable and valid for the Chinese population. Cronbach’s *α* coefficients of the HADS-A and HADS-D subscales were 0.753 and 0.764, respectively [18, 19].

The Fertility Problem Inventory (FPI) was developed by Newton et al. in 1999 [20]. The FPI is a 46-item self-rating scale assessing the level of infertility-related stress. Each item is scored on a 6-point Likert scale, ranging from 1 to 6. The FPI includes 5 dimensions: social concern (10 items), relationship concern (10 items), need for parenthood (10 items), rejection of childfree lifestyle (8 items), and sexual concern (8 items). Global stress is calculated by summing five subscale scores. The total scores range from 46 to 276. Higher scores indicate higher infertility-related stress. The Mandarin version of the FPI (M-FPI) was reliable and valid for Chinese infertile couples. The test–retest reliability of the FPI was 0.83 (female), and Cronbach’s *α* coefficient was 0.927 [5, 21].

### Primary and secondary outcomes

The primary outcome is the level of infertility-related stress measured by the Fertility Problem Inventory (FPI), before participants are discharged from the hospital. The secondary outcomes are the levels of anxiety and depression measured by the Hospital Anxiety and Depression Scale (HADS) before participants are discharged from the hospital.

### Data collection and management

The Hospital Anxiety and Depression Scale (HADS) and Fertility Problem Inventory (FPI) will be used to investigate the anxiety, depression and infertility-related stress of all participants at admission (t_0_) and at discharge (t_3_) from the hospital. The questionnaire will be administered via Wenjuanxing, the most popular web-based survey platform in China. Completeness checks were performed by allowing submission only after mandatory answering of all questions. Participants scan the QR code to receive the questionnaire. The platform promised to keep all data strictly confidential. Only researchers on this team (Huaxuan You and Anjiang Lei) will have access to the dataset. All the information will be coded without revealing personal data, and handled confidentially.

### Sample size

According to the pilot study, the standard deviations of FPI in the intervention group and control group were 22.91 and 29.01, respectively, and the means of FPI in the intervention group and control group were 144.29 and 159.31, respectively. We assumed that the number of patients in the intervention group was the same as that in the control group. The calculated sample size of 52 participants per group would provide 80% power with a two-tailed alpha error of 0.05. Estimating a dropout rate of approximately 5%, we will recruit at least 55 participants in each group.

### Randomization

The participants were assigned to the intervention group or control group by the random number table method. A computer-generated table of random numbers with parity matching was used. The obtained numbers were assigned to the continuously included participants. Participants with even numbers were assigned to the intervention group, and those with odd numbers were assigned to the control group. The generated random distribution sequence was placed in sequentially coded envelopes. After determining the eligibility of the participant, the researcher (You Huaxuan) opened the envelope and assigned the participant to the intervention group or control group.

### Blinding

Medical staff will be blinded to group allocation. The researcher (Chunyang Xi) who collected data will be blinded to group allocation. Other researchers and participants will be aware of the group allocation. The researcher who collected data will not be allowed to unblock the blinding.

### Statistical methods

The qualitative data (demographic characteristics) will be statistically described by number (*n*) and percentage (%) and statistically be inferred by chi-square test. The Shapiro–Wilk test will be used to assess the normality of our data. This test is suitable for small to medium-sized samples and will help us determine whether the data follows a normal distribution. The quantitative data (scores of HADS and FPI) will be described by mean/median and standard deviation (SD)/interquartile range (IQR) and be inferred by *t* test/rank sum test. In all analyses, a *P* value of < 0.05 indicates statistical significance. To handle protocol non-adherence in our randomized controlled trial, we will use an intention-to-treat (ITT) analysis. Additionally, we will conduct a per-protocol (PP) analysis to include only those participants who fully adhered to the study protocol. By comparing ITT and PP results, we aim to understand the impact of adherence on study outcomes. All instances of non-adherence will be documented and considered during data analysis and interpretation. SPSS 21.0 (SPSS Inc., Chicago, IL) will be used for statistical analysis.

### Data monitoring

All the interventions applied in this study have been used in clinical practice and are not potentially harmful, so the data monitoring committee is not included in this study. Any unexpected adverse events that occurred during the intervention period will be reported to an experienced psychologist who will not be involved in this trial. To ensure the safety and effectiveness of the study, we will conduct an interim analysis when approximately 50% of the participants have completed the trial.

### Ethics approval and consent to participate

This study was approved by the ethics committee of West China Second University Hospital (No. 2022283). All amendments proposed by the ethics committee were sent to the ethics committee before study approval. One researcher (Anjiang Lei) will obtain the informed consent from all participants. All data provided to our study will be kept in strict confidence, and none of the participants’ identifying information will be reported. All procedures in this study will be conducted in accordance with the ethical standards of the responsible committee on human study and with the Helsinki Declaration and later revision.

### Dissemination plans

The results of this study will be published in a scientific journal. The selection of the authorship is based on several considerations, including the following items: contribute to the study design, data collection, data analysis, data interpretation, manuscript drafting, and manuscript revising. Scientific presentations for researchers, healthcare practitioners, and/or the public present another forum for dissemination of our results.

## Discussion

Infertility has several health and social consequences, including poor physical and mental well-being, disease stigma, violence from family, marriage breakdown, and economic pressure [22]. Infertility affects the mental well-being of more women than men. Infertile women had higher levels of infertility-related stress, anxiety, and depression than infertile men [5, 8]. Infertile women coping with stress might benefit by disclosing their experience of infertility, so mental care needs to encourage the expression and sharing of emotions during treatment [11, 12]. Expressive art therapy (EAT) has been incorporated into various programs as an element of both foundational and supportive treatment. EAT combines physical and psychological nonmainstream methods, including dance/movement, drawing, painting, sculpting, music, writing, sound, and improvisation [13]. This diversity of intervention methods caters to the varied needs of patients, providing a more comprehensive and adaptable treatment framework. Moreover, this study underscores the significance of cultural sensitivity among expressive art therapists, which is instrumental in effectively engaging patients from Chinese cultural backgrounds, thus enhancing the efficacy of interventions.

This study will employ a randomized controlled trial design, bolstering internal validity. Randomly assigning participants to either the intervention or control groups serves to mitigate the impact of selection bias, heightening the reliability of our results [23]. Additionally, the study will use robust measurement tools such as the Hospital Anxiety and Depression Scale (HADS) and the Fertility Problem Inventory (FPI) for assessing anxiety, depression, and infertility-related stress. These instruments have demonstrated validity and reliability in prior research, ensuring the robustness and comparability of our findings [18, 19, 21].

Thus, our study will verify the effectiveness of EAT for anxiety, depression, and infertility-related stress in infertile women. If our hypothesis is supported, this study will provide valuable information on mental health care strategies for infertile women and inform the design of future research and programs focused on infertility-related stress interventions.

## Trial status

The study protocol was version 1.0 on the date of March 2023 for ethical review in the West China Second University Hospital. Recruitment commenced in June 2023. Recruitments will be completed in December 2023.

### Supplementary Information

Supplementary Material 1.

## Data Availability

Data will be available from the corresponding author upon request once they have been collected.

## Abbreviations

HADS: Hospital Anxiety and Depression Scale
FPI: Fertility Problem Inventory
EAT: Expressive art therapy
SPIRIT: Standard Protocol Items: Recommendations for Interventional Trials
QR code: Quick Response Code
SD: Standard deviation
IQR: Interquartile range
